# Assessing the reporting quality of adult neuro-oncology protocols, abstracts, and trials: Adherence to the SPIRIT and CONSORT statements

**DOI:** 10.1093/nop/npad017

**Published:** 2023-03-22

**Authors:** Joshua S Suppree, Avni Patel, Sumirat M Keshwara, Sandhya Trichinopoly Krishna, Conor S Gillespie, George E Richardson, Mohammad A Mustafa, Sophia Hart, Abdurrahman I Islim, Michael D Jenkinson, Christopher P Millward

**Affiliations:** School of Medicine, University of Liverpool, Liverpool, United Kingdom; Department of Neurosurgery, The Walton Centre NHS Foundation Trust, Liverpool, United Kingdom; School of Medicine, University of Liverpool, Liverpool, United Kingdom; Department of Neurosurgery, The Walton Centre NHS Foundation Trust, Liverpool, United Kingdom; Department of Neurosurgery, The Walton Centre NHS Foundation Trust, Liverpool, United Kingdom; School of Medicine, University of Liverpool, Liverpool, United Kingdom; Department of Neurosurgery, The Walton Centre NHS Foundation Trust, Liverpool, United Kingdom; School of Medicine, University of Liverpool, Liverpool, United Kingdom; Department of Neurosurgery, The Walton Centre NHS Foundation Trust, Liverpool, United Kingdom; Department of Neurosurgery, The Walton Centre NHS Foundation Trust, Liverpool, United Kingdom; School of Medicine, University of Liverpool, Liverpool, United Kingdom; Department of Neurosurgery, The Walton Centre NHS Foundation Trust, Liverpool, United Kingdom; Department of Neurosurgery, The Walton Centre NHS Foundation Trust, Liverpool, United Kingdom; Institute of Systems, Molecular, and Integrative Biology, University of Liverpool, Liverpool, United Kingdom; Department of Neurosurgery, The Walton Centre NHS Foundation Trust, Liverpool, United Kingdom; Institute of Systems, Molecular, and Integrative Biology, University of Liverpool, Liverpool, United Kingdom; Department of Neurosurgery, The Walton Centre NHS Foundation Trust, Liverpool, United Kingdom; Institute of Systems, Molecular, and Integrative Biology, University of Liverpool, Liverpool, United Kingdom

**Keywords:** clinical trial, CONSORT-A, CONSORT, SPIRIT

## Abstract

**Background:**

Comprehensive and transparent reporting of clinical trial activity is important. The Standard Protocol Items: Recommendations for Interventional Trials (SPIRIT) 2013 and Consolidated Standards of Reporting Trials (CONSORT) 2010 statements define the items to be reported in clinical trial protocols and randomized controlled trials, respectively. The aim of this methodological review was to assess the reporting quality of adult neuro-oncology trial protocols and trial result articles.

**Methods:**

Adult primary and secondary brain tumor phase 3 trial protocols and result articles published after the introduction of the SPIRIT 2013 statement, were identified through searches of 4 electronic bibliographic databases. Following extraction of baseline demographic data, the reporting quality of independently included trial protocols and result articles was assessed against the SPIRIT and CONSORT statements respectively. The CONSORT-A checklist, an extension of the CONSORT 2010 statement, was used to specifically assess the abstract accompanying the trial results article. Percentage adherence (standard deviation [SD]) was calculated for each article.

**Results:**

Seven trial protocols, and 36 trial result articles were included. Mean adherence of trial protocols to the SPIRIT statement was 79.4% (SD: 0.11). Mean adherence of trial abstracts to CONSORT-A was 75.3% (SD: 0.12) and trial result articles to CONSORT was 74.5% (SD: 0.10).

**Conclusion:**

The reporting quality of adult neuro-oncology trial protocols and trial result articles requires improvement to ensure comprehensive and transparent communication of planned neuro-oncology clinical trials and results within the literature. Raising awareness by clinical triallists and implementing mandatory evidence of proof of adherence by journals should improve reporting quality.

Clinical trials are designed to investigate the comparative effectiveness (superiority or noninferiority of a therapeutic option against another) in order to allow new treatment recommendations to be made.^1^ Randomized controlled trials (RCTs) are generally regarded as the “gold standard” methodological approach, by isolating the influence of the intervention in question on outcome, through a process of randomization to treatment arm.^2^ However, without comprehensive and transparent reporting of the planned methods (trial protocol) and results (trial results abstract and article), critical review and comparative analysis may be compromised. To this end, efforts have been made to standardize the reporting of these components with the publication of statements that describe the items to be reported on.

The Standard Protocol Items: Recommendations for Interventional Trials (SPIRIT) 2013 guidance provides evidence-based recommendations for the minimum items that should be included when drafting a clinical trial protocol for an interventional clinical trial.^3^ The checklist includes 51 items. Adherence to SPIRIT ensures that all critical methodological aspects of the design of a clinical trial are addressed a priori.

The Consolidated Standards of Reporting Trials (CONSORT) statement, most recently updated in 2010, is an evidence-based, minimum set of recommendations for the reporting of randomized trials^4^ and is endorsed by over 600 medical journals.^5,6^ The statement includes 25 items focused on reporting how a trial was designed, analyzed, and interpreted, with the overall aim of improving transparency of trial reporting. There are extensions of the CONSORT statement, notably CONSORT-A, which details specific considerations for the reporting of trial abstract items.^7^

Taken together, the SPIRIT and CONSORT statements offer globally recognized reporting guidance to clinical triallists seeking to effectively communicate their planned and completed RCT. If a trial protocol or trial results article is not reported to these standards, the ability of a trial to inform clinical decision-making could be hampered. This creates difficulty, not only for the clinical triallist conducting the study, but for those seeking to interpret its results, for instance, other investigators including researchers, patients, funders and sponsors, ethics committees and institutional review boards, trial registries, and policymakers/regulators.^3^ The simplicity of a checklist allows both the author and anyone critiquing the work, to identify these items of importance.

The standard of reporting quality in adult neuro-oncology trial protocols and clinical trial results has not been assessed to date. Therefore, the aim of this review was to assess the reporting quality of adult, phase 3 neuro-oncology trial protocols and trial result articles (concerning adult primary and secondary brain tumors) published since 2014 onwards, in line with the SPIRIT statement introduction in 2013 and the CONSORT statement update in 2010. In doing so, we aim to raise awareness of these tools, for both clinical triallists, and those seeking critical review of the neuro-oncology clinical trial literature.

## Material and Methods

### Information Sources

A detailed search strategy was developed to identify adult, phase 3 neuro-oncology RCTs from the published literature, since 2014. The following electronic bibliographic databases were searched: PubMed, EMBASE, CINAHL, and the Cochrane central register of controlled trials. The complete search strategies are provided in Supplementary Appendix 1.

### Eligibility Criteria

For the purposes of this methodological review, a sample of neuro-oncology clinical trial protocols and clinical trial result articles was required for subsequent analysis. The 2 article types did not need to be linked as pertaining to the same study and were evaluated separately. Therefore, the inclusion criteria for eligible studies were specified as published clinical trial protocols and clinical trial result papers (concerning phase 3 RCTs), that describe cohorts of adults (minimum 10 patients) with an intracranial tumor (glial, meningioma, or cerebral metastases), receiving interventions including perioperative care, surgery, radiotherapy, pharmacotherapy, or any combination of the above. Only full-text articles written in the English language (due to limitations of translation services) and published after January 1, 2014 were eligible for inclusion. The full eligibility criteria are provided in Supplementary Appendix 2.

### Study Selection and Data Extraction

Search results were downloaded from their respective online databases, and a file for each was uploaded to the online platform Rayyan,^8^ for the purposes of deduplication and screening. All potentially eligible titles and abstracts were screened by 2 review authors (J.S.S. and A.P.). For those appearing to meet the eligibility criteria, or for those where a decision could not be made based on title and abstract alone, full-text copies were obtained. All full-text articles were then screened against the eligibility criteria by the same 2 review authors (J.S.S. and A.P.). Any titles which did not achieve concordance were highlighted within the platform, discussed, and resolved between the 2 review authors in person or escalated to the senior review author (C.P.M.).

Data were extracted independently from all eligible articles into a custom-designed and piloted data extraction spreadsheet in Microsoft Excel by 2 review authors (J.S.S. and A.P.). The first 10% of included articles were cross-checked for concordance, and if less than 5% variation in extracted data existed, data extraction proceeded without question.

The following study demographic data were extracted: Surname and country of first author, year, and journal of publication plus associated impact factor (as of 2021), protocol or trial title, phase intervention being carried out, and tumor type studied. In cases where multiple tumors were studied, the primary tumor type was recorded.

### Assessment of Reporting Quality

Reporting quality of trial protocols was assessed against the SPIRIT 2013 statement checklist which has 51 items (Supplementary Appendix Table 3).^9^ A single point was awarded for adequate reporting of an item in the manuscript or supplementary material, as judged by the review author (Yes = 1 point, No = 0 point). The maximum score was 51 as all items on the SPIRIT checklist were applicable to protocols eligible for inclusion in this review.

Reporting quality of included clinical trial result articles were assessed against the CONSORT-A and CONSORT statement checklists. CONSORT-A checklist included 17 items (Supplementary Appendix 4) and CONSORT checklist included 37 items (Supplementary Appendix 5).

A single point was awarded for adequate reporting of an item in the manuscript or supplementary material, as judged by the review author (Yes = 1 point, No = 0 point). For studies where an item on the checklist was not applicable (N/A), the maximum score attainable for that paper was reduced by one point. This was to remove penalization for non-applicable requirements. For example, item 11b “Similarity of interventions—If relevant, description of the similarity of interventions” was only applicable if the trial had 2 different interventions which were deemed similar in comparison to one another. If the trial had 2 interventions that were deemed dissimilar item 11b would receive N/A for that trial.^10^ Additionally, item 14b on the CONSORT checklist “Why the trial ended or was stopped” was only applicable for trials that ended or stopped before their natural conclusion.^10^ In conjunction with the accompanying explanation and elaboration document, if the trial was not ended or stopped early the trial would receive N/A for that question on the checklist. Furthermore, item 17b “For binary outcomes, presentation of both absolute and relative effect sizes is recommended”^10^ was only relevant for trials with binary outcomes. In this review, none of the trials had binary outcomes so all trials received N/A for this item on the checklist.

### Statistical Analysis

Descriptive analysis was used to calculate the proportion (shown as a percentage) of SPIRIT statement items that were adequately reported in protocols. Mean values are presented alongside the standard deviation (SD). The same analysis was carried out on the clinical trial results from articles using the CONSORT-A and CONSORT 2010 statement checklists. Analysis was carried out on Microsoft Excel.

## Results

### Study Characteristics

**Figure 1.**
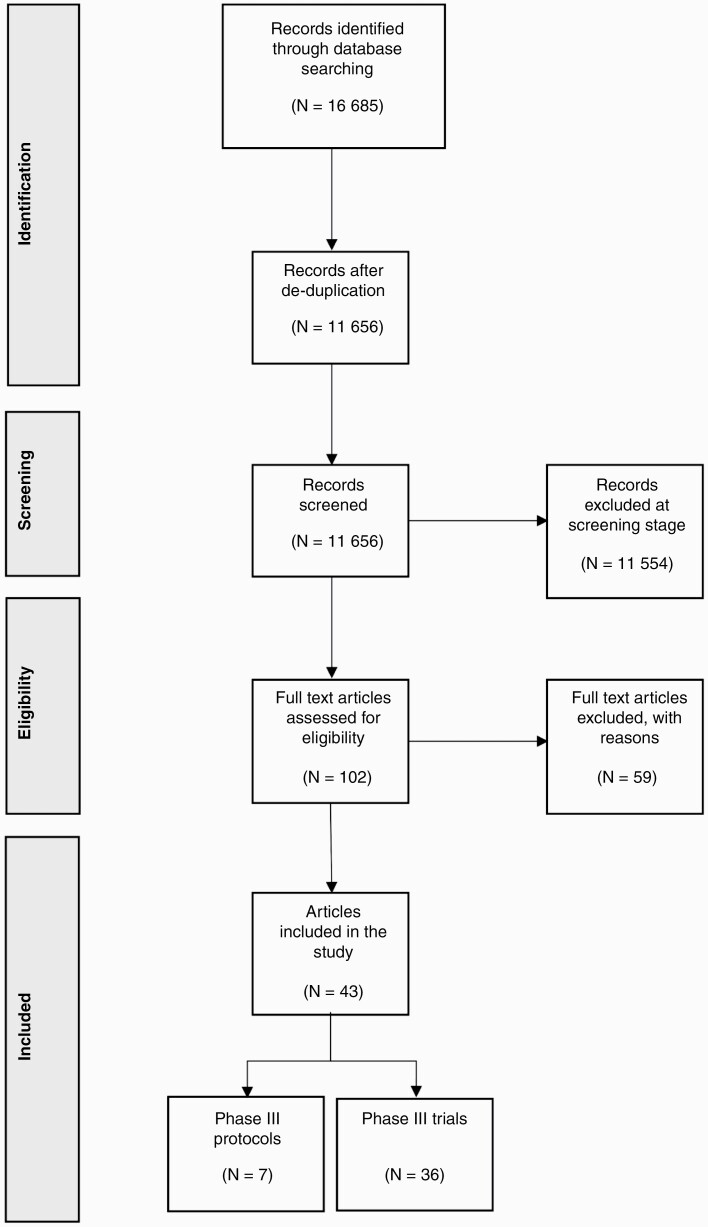
Preferred Reporting Items for Systematic Reviews and Meta-Analyses (PRISMA) Flow Chart – 16 685 articles identified following initial search. Screening of titles and abstracts identified 102 articles for full-text review. Forty-three articles were included in the study.

Forty-three articles were included in this review, of which 7 were trial protocols and 36 were phase 3 clinical trial result articles. The included trial protocols and phase 3 clinical trial result articles were independent of one another. The search, screening, and selection results are summarized in Figure 1. Most of the included protocols described planned phase 3 trials, apart from a single study that related to a phase 1/3 trial. Of the included protocols, 43% (*n* = 3) had a first author affiliated with an institution in North America. Publication in BMC Cancer, which endorses the use of the SPIRIT statement, accounted for 43% (*n* = 3) of the protocols.^11^Table 1 provides an overview of the included protocols.

**Table 1.** Overview of included protocols assessed using SPIRIT statement

	1^st^ Author	Country	Year	Journal	Trial name	Trial phase	Tumor type
1	Michael D Jenkinson	UK	November 2014	*Trials*	The ROAM/EORTC-1308 trial: Radiation vs. Observation following surgical resection of Atypical Meningioma	3	Atypical Meningioma
2	Michelle J Naughton	USA	May 2018	*Neuro Oncology Practice*	Quality of life of irradiated brain tumor survivors treated with donepezil or placebo: Results of the WFU CCOP research base protocol 91105	3	Any primary or metastatic brain tumor
3	Georgia K B Halkett	Australia	October 2015	*BMJ Open*	Protocol for the Care-IS Trial: A randomized controlled trial of a supportive educational intervention for carers of patients with high-grade glioma (HGG)	3	High-Grade Glioma
4	Robert Olson	Canada	May 2020	*BMC Cancer*	Stereotactic ablative radiotherapy for the comprehensive treatment of 1–3 Oligometastatic tumors (SABR-COMET-3): Study protocol for a randomized phase III trial	3	Oligometastatic tumors
5	David A Palma	Canada	August 2019	*BMC Cancer*	Stereotactic ablative radiotherapy for the comprehensive treatment of 4–10 oligometastatic tumors (SABR-COMET-10): Study protocol for a randomized phase III trial	3	Oligometastatic tumors
6	Lin Kong	China	February 2019	*Cancer Communications London*	Carbon ion radiotherapy boost in the treatment of glioblastoma: A randomized phase I/III clinical trial	Phase 1/3	Glioblastoma
7	Jaap D Zindler	Netherlands	July 2017	*BMC Cancer*	Whole brain radiotherapy versus stereotactic radiosurgery for 4–10 brain metastases: a phase III randomized multicentre trial	3	4–10 Brain Metastases

Over half of the clinical trial result articles had a first author affiliated with an institution in the United States (53%, *n* = 19), while 25% (*n* = 9) were affiliated with an institution in Europe, and the remainder from the rest of the world 22% (*n* = 8). Publication in the Journal of Clinical Oncology accounted for 36% (*n* = 13) of the clinical trial articles, while 25% (*n* = 9) of trials were published by the Lancet publication group, including the Lancet and Lancet Oncology. Glioblastoma was the study subject for 42% (*n* = 15). Table 2 provides an overview of the included clinical trial articles.

**Table 2.** Overview of included phase III trials and abstracts assessed using the CONSORT 2010 statement and CONSORT-A checklist

	First Author	Country	Year	Journal	Trial Name	Trial Phase	Tumor Type
1	Wellar, M	Switzerland	August 22, 2017	*The Lancet Oncology*	Rindopepimut with temozolomide for patients with newly diagnosed, EGFRvIII-expressing glioblastoma (ACT IV)	3	Glioblastoma
2	Angela M. Hong	Australia	Novemebr 20, 2019	*Journal of Clinical Oncology*	Adjuvant Whole-Brain Radiation Therapy Compared With Observation After Local Treatment of Melanoma Brain Metastases	3	Melanoma Metastases
3	Florence Laigle-Donadey	France	May–December 2019	*Neuro-oncology advanced*	study of dexamphetamine sulfate for fatigue in primary brain tumors patients: An ANOCEF trial (DXA)	3	Primary brain tumor
4	Olivier L Chinot	France	2014	*The New England Journal of Medicine*	bevacizumab plus radiotherapy-temozolomide for newly diagnosed glioblastoma	3	Glioblastoma
5	J. Gregory Cairncross	Canada	2013	*Journal of Clinical Oncology*	Chemotherapy plus radiotherapy (CT-RT) versus RT alone for patients with anaplastic oligodendroglioma	3	anaplastic oligodendroglioma
6	Roger Stupp	USA	August 19, 2014	*The Lancet Oncology*	Cilengitide combined with standard treatment for patients with newly diagnosed glioblastoma with methylated MGMT promoter	3	Glioblastoma
7	Yongli Ji	USA	December 2015	*Journal of Clinical Oncology*	Double-Blind Phase III Randomized Trial of the Antiprogestin Agent Mifepristone in the Treatment of Unresectable Meningioma	3	Unresectable Meningioma
8	Jan C. Buckner	USA	May 2014	*journal of Clinical Oncology*	Phase III study of radiation therapy (RT) with or without procarbazine, CCNU, and vincristine (PCV) in low-grade glioma: RTOG 9802 with Alliance, ECOG, and SWOG.	3	Low-Grade Glioma
9	Erica H Bell	USA	July 24, 2020	*Journal of Clinical Oncology*	Radiation Versus Radiation Plus Procarbazine, Lomustine (CCNU), and Vincristine in High-Risk Low-Grade Glioma	3	Low-Grade Glioma
10	Mark R. Gilbert	USA	October 07, 2013	*Journal of Clinical Oncology*	Dose-dense temozolomide for newly diagnosed glioblastoma	3	Glioblastoma
11	Yongli Ji	USA	December 1, 2015	*Journal of Clinical Oncology*	Antiprogestin Agent Mifepristone in the Treatment of Unresectable Meningioma: SWOG S9005	3	Meningioma
12	David A. Reardon	USA	July 2020	*JAMA oncology*	Effect of Nivolumab vs. Bevacizumab in Patients With Recurrent Glioblastoma	3	glioblastoma
13	Yoshitaka Narita	Japan	February 2019	*Neuro-Oncology*	Trial of personalized peptide vaccination for recurrent glioblastoma	3	Glioblastoma
14	D. A. Reardon	USA	May 2020	*JAMA Oncology*	Effect of Nivolumab vs. Bevacizumab in Patients With Recurrent Glioblastoma	3	Glioblastoma
15	Johannes Weller	Germany	October 2019	*The Lancet Oncology*	Health-related quality of life and neurocognitive functioning with lomustine-temozolomide vs. temozolomide in patients with newly diagnosed, MGMT-methylated glioblastoma (CeTeG/NOA-09)	3	Glioblastoma
16	Paul D Brown	USA	April 2020	*Journal of Clinical Oncology*	Hippocampal Avoidance During Whole-Brain Radiotherapy Plus Memantine for Patients With Brain Metastases	3	Any Brain Metastases
17	Jan Buckner	USA	Novemebr 2015	*Neuro-Oncology*	Radiation therapy (RT) alone vs. RT plus procarbazine, ccnu, and vincristine (PCV) in patients with low-grade glioma (LGG)	3	Low-Grade Glioma
18	Martin J van den Bent	Netherlands	August 2017	*The Lancet*	CATNON trial (EORTC study 26053-22054) of treatment with concurrent and adjuvant temozolomide for 1p/19q non-co-deleted anaplastic glioma	3	Anaplastic Glioma
19	Wilson Roa	Canada	September 2015	*Journal of Clinical Oncology*	International Atomic Energy Agency Study of Radiation Therapy in Elderly and/or Frail Patients With Newly Diagnosed Glioblastoma Multiforme	3	Glioblastoma
20	Takamasa Kayama	Japan	May 2016	*Journal of Clinical Oncology*	JCOG0504: Surgery with whole brain radiation therapy versus surgery with salvage stereotactic radiosurgery in patients with 1 to 4 brain metastases.	3	Any Brain Metastases
21	Ulrich Herrlinger	Germany	February 2019	*The Lancet*	Lomustine-temozolomide combination therapy versus standard temozolomide therapy in patients with newly diagnosed glioblastoma with methylated MGMT promoter	3	Glioblastoma
22	Roger Stupp	USA	December 2015	*JAMA oncology*	Maintenance Therapy With Tumor-Treating Fields Plus Temozolomide vs. Temozolomide Alone for Glioblastoma	3	Glioblastoma
23	P. Navarria	Italy	September 2016	*Neuro-Oncology*	Randomized double arm phase III study to evaluate feasibility and safety of Gamma Knife radiosurgery versus Linac Based (Edge) radiosurgery in brain metastatic patients	3	Any Brain Metastases (UP TO 4)
24	Manfred Westphal	Germany	January 2015	*European Journal of Cancer*	trial with nimotuzumab, an anti-epidermal growth factor receptor monoclonal antibody in the treatment of newly diagnosed adult glioblastoma	3	Glioblastoma
25	Anita Mahajan	USA	July 2014	*The Lancet Oncology*	Post-operative stereotactic radiosurgery vs. observation for completely resected brain metastases	3	Any Brain Metastases
26	David Roberge	Canada	November 2017	*Neuro-Oncology*	POST-OPERATIVE RADIOSURGERY COMPARED WITH WHOLE BRAIN RADIOTHERAPY FOR RESECTED METASTATIC BRAIN DISEASE: COGNITIVE FUNCTION OF LONG-TERM SURVIVORS	3	Any Brain Metastases
27	Paul D Brown	USA	July 2017	*The lancet Oncology*	Post-operative stereotactic radiosurgery compared with whole brain radiotherapy for resected metastatic brain disease	3	any Brain Metastases
28	Paul D Brown	USA	June 2015	*Journal of Clinical Oncology*	Whole brain radiation therapy (WBRT) in addition to radiosurgery (SRS) in patients with 1 to 3 brain metastases	3	Any Brain Metastases
29	Terri S Armstrong	USA	October 2013	*Journal of Clinical Oncology*	Trial comparing conventional adjuvant temozolomide with dose-intensive temozolomide in patients with newly diagnosed glioblastoma	3	Glioblastoma
30	Paul D Brown	USA	July 2014	*The Lancet ­Oncology*	Post-operative stereotactic radiosurgery compared with whole brain radiotherapy for resected metastatic brain disease	3	Any Brain Metastases
31	Ulrich Herrlinger	Germany	February 2019	*The Lancet*	Lomustine-temozolomide combination therapy versus standard temozolomide therapy in patients with newly diagnosed glioblastoma with methylated MGMT promoter (CeTeG/NOA–09): a randomized, open-label, phase 3 trial	3	Glioblastoma
32	Arif N Ali	USA	February 2018	*Journal of Neuro-oncology*	Trial of hyperfractionated radiotherapy (RT) and BCNU vs. standard RT and BCNU for malignant glioma patients	3	Glioma
33	Doo-Sik Kong	Korea	January 2017	*Oncotarget*	Autologous cytokine-induced killer cell immunotherapy for newly diagnosed glioblastoma in Korea	3	Glioblastoma
34	Jan C. Buckner	USA	May 2014	*Journal of Clinical Oncology*	Phase III study of radiation therapy (RT) with or without procarbazine, CCNU, and vincristine (PCV) in low-grade glioma: RTOG 9802 with Alliance, ECOG, and SWOG.	3	Low-Grade Glioma
35	Susan Chang	USA	February 2017	*Neuro-Oncology*	Radiation and temozolomide vs. radiation and nitrosourea therapy for anaplastic astrocytoma	3	Anaplastic Astrocytoma
36	Deborah T Blumenthal	Israel	November 20s14	*International Journal of Clinical Oncology*	radiation therapy (RT) and O⁶-benzylguanine + BCNU versus RT and BCNU alone and methylation status in newly diagnosed glioblastoma and gliosarcoma	3	Glioblastoma and Gliosarcoma

### Quality of Reporting as Per SPIRIT 2013 Statement

Seven protocols were included in this review and assessed against the SPIRIT statement. An average adherence rate of 79.4% (SD: 0.11) was observed. The range of compliance with the 51 items in the checklist was 32/50 to 46/50. There was one “non-applicable” question in the SPIRIT statement regarding item 33 “biological specimens.” If a protocol was not collecting biological specimens for analysis it would not be applicable for that protocol to explain the methods used to store such specimens. All included protocols reported the administrative information for the protocol, described the background and rationale, and reported the study setting. Only 57.1% (*n* = 4) of the protocols included explained the choice of comparators.

Although all 7 protocols described the planned interventions in each group, including administration methods, only 71.4% (*n* = 5) of protocols described criteria for discontinuing or modifying the allocated interventions as well as strategies to improve protocol adherence. Furthermore, only one protocol listed concomitant care and interventions that would be allowed or prohibited throughout the trial. The assignment of interventions, including details on sequence generation, was reported in 57.1% (*n* = 4) of protocols and allocation and implementation in only 42.9% (*n* = 3). Only 28.6% (*n* = 2) of protocols reported whether blinding took place.

**Figure 2.**
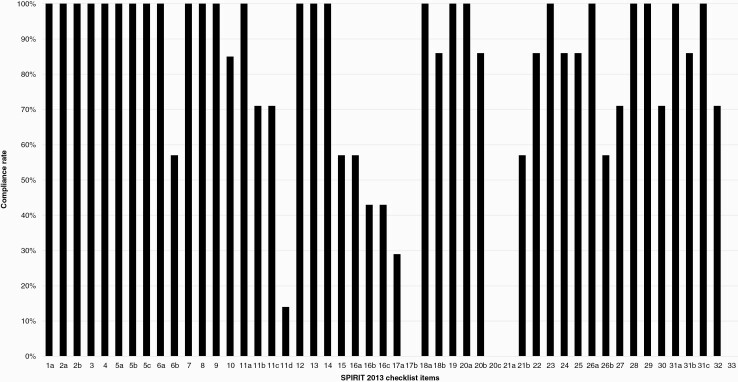
Compliance rate (%) to SPIRIT 2013 statement. Note that item 17c was deemed “not applicable” to any of the included protocols.

All 7 protocols reported planned statistical analysis and a further 85.7% (*n* = 6) of trials described any planned additional analyses. However, all protocol authors failed to report details regarding protocol nonadherence and any statistical methods to handle missing data in the population analyzed. A summary of the adherence rates for each item in the SPIRIT 2013 checklist can be seen in Figure 2 and in Supplementary Appendix 6.

### Quality of Reporting as per CONSORT-A checklist

Thirty-six clinical trial abstracts from the results article were assessed against the CONSORT-A statement. Average adherence rate with the checklist was 75.3% (SD: 0.12). The range of compliance with items from the checklist was 6/17 to 15/17. All items on the CONSORT-A checklist were deemed applicable to every abstract. Randomization was identified in the title in 80.5% (*n* = 29) of trial abstracts, and corresponding authors' details and trial design were reported in 88.8% (*n* = 32) of included abstracts. All abstracts (*n* = 36) accurately reported the trial objectives, interventions, and outcomes.

Randomization, including the strategy to allocate participants to interventions, was only reported in 38.9% (*n* = 14) of abstracts, and information on blinding was reported in only 27.8% (*n* = 10) of abstracts. Trial status was reported in 44% (*n* = 16) of included abstracts.

**Figure 3.**
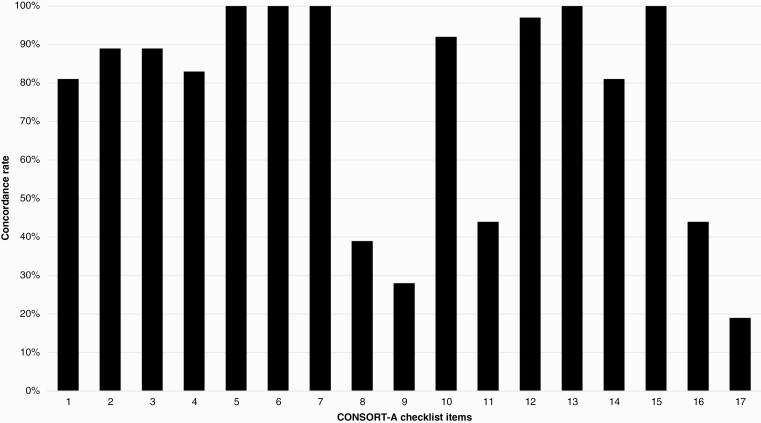
Compliance rate (%) to CONSORT-A Checklist.

Primary outcome, estimated effect size and precision, conclusions, and result interpretation were reported accurately in all abstracts. Details on trial registration were reported in only 38.9% (*n* = 14) of the included trial abstracts and only 27.7% (*n* = 10) mentioned trial funding. A summary of the compliance rates of each item on the CONSORT-A checklist can be seen in Figure 3 below and in Supplementary Appendix 7.

The included abstracts were also analyzed based on their year of publication. A two-sample *t*-test was performed to compare trials published in 2013–2017 (group 1, *n* = 25) to trials published between 2018 and 2022 (group 2, *n* = 11). There was no significant difference in concordance rate (%) between group 1 (mean = 75.6%, SD: 0.10) and group 2 (mean = 74.3, SD: 0.16), *P* = .76.

### Quality of Reporting as per CONSORT 2010 Statement

Thirty-six phase 3 RCTs were included in this analysis. After accounting for non-applicable items, the mean score was 74.5% (SD: 0.10) with a range of 22/34 to 31/34. Identification of the trial as randomized in the title was present in 80.5% (*n* = 29). All included trials discussed the scientific background of their paper and highlighted any objectives or hypotheses clearly. 88.8% (*n* = 32) of trials discussed the trial design, including allocation ratio, with points only being awarded if both the allocation ratio and design were mentioned. Only 13.9% (*n* = 5) of trials discussed any important changes that were made after the trial commenced. If no changes were made but the trial explicitly stated this, then they would also receive a “Yes.” All trials described primary and secondary outcome measures, including how these measures would be assessed, but no included trials reported whether changes had been made to the objectives after the trial had commenced.

Reporting on randomization methods was varied across the included RCTs. While 80.5% (*n* = 29) of studies identified the trial as randomized in the title, only 69.4% (*n* = 25) described the method used to generate the randomization sequence in the study. Furthermore, only 22.2% (*n* = 8) trials described the implementation of randomization, including who generated the randomized allocation sequence, who enrolled the participants, and who assigned the interventions. Information on blinding was also inadequate, only being reported in 25% (*n* = 9) of studies.

For 91.2% (*n* = 33) of included trials, the item questioning the similarity of interventions was deemed not applicable because many trials had interventions that were not comparable. All of the included trials did not include binary outcomes, hence all received N/A for this item against the CONSORT checklist.

**Figure 4.**
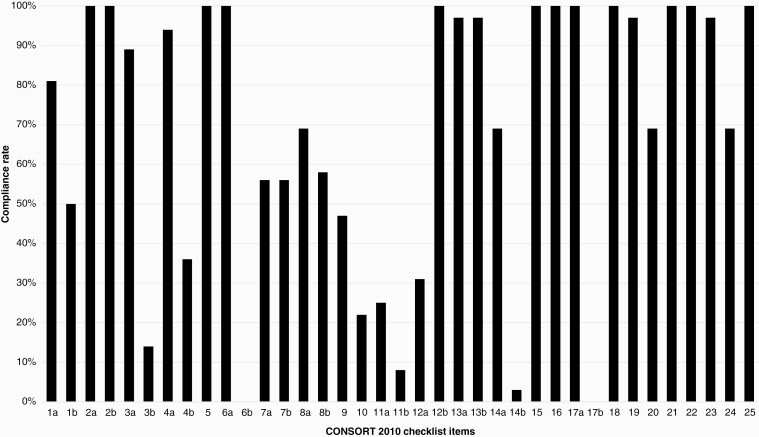
Compliance rate (%) to CONSORT 2010 Statement Checklist. Note item 17b was deemed “not-applicable” to any of the included trials.

Trial generalizability was reported in all included trials. However, the trial limitations, including sources of bias and misinterpretation were reported in only 69.4% (*n* = 25) of included trials. All articles (*n* = 36) included descriptions of funding and the trial registration number. Only 69.4% (*n* = 25) reported where the full trial protocol could be found. A summary of compliance rate can be seen in Figure 4 below and in Supplementary Appendix 8.

The included phase III trials were also analyzed based on the year of publication. A two-sample *t*-test was performed to compare trials published in 2013–2017 (group 1, *n* = 25) to trials published between 2018 and 2022 (group 2, *n* = 11). There was no significant difference in concordance rate (%) between group 1 (mean = 75.5%, SD: 0.08) and group 2 (mean = 72.0%, SD: 0.12), *P* = .31. It was also investigated whether the journal where a phase III trial was published in influenced the reporting quality. A Pearson correlation coefficient was computed to assess for a linear relationship between journal impact factor and the concordance rate (%) to the CONSORT checklist. There was a small positive correlation between the 2 variables, *r* = 0.28 (*n* = 36). However, this relationship was not statistically significant (*P* = .10).

### Summary of Key Results

The quality of reporting as per SPIRIT 2013 statement displayed inadequate reporting of the assignment of interventions, with details on sequence generation being reported in only 57.1% (*n* = 4) protocols and allocation and implementation in only 42.9% (*n* = 3). Blinding was reported in 28.6% (*n* = 2) of the included protocols. All abstracts (*n* = 36) accurately reported the trial objectives, interventions, and outcomes using the CONSORT-A statement. Randomization, including the strategy to allocate participants to interventions, was only reported in 38.9% (*n* = 14) of abstracts and information on blinding was reported in only 27.8% (*n* = 10) of abstracts. The quality of reporting as per CONSORT 2010 statement was 100% (*n* = 36) when trials discussed the scientific background to their paper and highlighted any objectives or hypothesis clearly. However, only 69.4% (*n* = 25) described the method used to generate the randomization sequence in the study. Trial limitations, including sources of bias and misinterpretation, were reported in only 69.4% (*n* = 25) of included trials.

## Discussion

RCTs are the “gold standard” methodological tool used to provide evidence of comparative effectiveness, and established guidance exists to facilitate the reporting of both trial protocols and clinical trial results. This is the first analysis of the quality of reporting of adult neuro-oncology clinical trial protocols and clinical trial results. The study highlights the common reporting deficiencies.

### Trial Protocols and the SPIRIT 2013 Statement

The SPIRIT 2013 statement serves as a framework of important items to include in a clinical trial protocol, and to facilitate comprehensive and transparent reporting. Administrative information including the title, registration, protocol version, funding, and responsibilities was found to be reported in the included protocols. All protocols also provided a description of funding sources so the reader can objectively assess and evaluate any potential conflicting interests.^3^

Almost all protocols described the research question and justified the need for the trial, however not all protocols explained the choice of comparators. Only 42.9% (*n* = 3) of protocols included reasoning for comparators, highlighting an area where improvements can be made in future protocols.

All protocols reported the trial design in sufficient detail to enable replication. This is important in order to provide a context for a protocol and to ensure participants in the study are appropriate, fulfill certain specifications, and are representative of the target population. Many protocols did not discuss strategies to improve adherence to intervention protocols, and any procedures for monitoring adherence.^3^ Relevant concomitant care and interventions that are permitted or prohibited during the trial were very poorly reported, with only one protocol discussing it. Concomitant care is an important element of any protocol as there should only be one variable intervention between the trial and control groups and concomitant care can act as a confounding variable or co-intervention if not properly controlled for.^3^

Randomization details were poorly reported in the included protocols. Randomization is a cornerstone of the study design and specific standards must be upheld when reporting the associated methodology.^12^ Only a minority of protocols adequately reported the methods and mechanisms used to generate the randomized allocation sequence. Failing to report this information can hinder a reader's ability to measure the magnitude and precision of the treatment effect, as well as any selection bias.^13^ Similarly, only a small proportion of protocols adequately reported details on blinding. When reporting prospective clinical trial protocols efforts should be made to report succinct and compressive details on the randomization process and, if applicable, blinding.

Methodological processes, including description of study setting, as well as eligibility criteria were reported particularly well throughout the included protocols. Many protocols also reported ways to promote participant retention and complete follow-up which is important to maximize completeness of data collection.^3^ Statistical methods used to analyze data were described in most protocols, with justifications for choice of statistical methods and any comparisons which had been made. There was very poor compliance with reporting on any instances of protocol nonadherence, which allows speculation regarding possible missing data which might have an impact on the outcomes reported. Prospective clinical trial protocols should make effort to comment on how they will manage protocol nonadherence and remove speculation from the reader.

Ethical approval and important trial modifications are adequately reported in most protocols. Ethical approval is a universal requirement in clinical research,^3^ however for completeness all protocols should report how they applied for approval, the granting body and declare all protocol amendments post ethical approval.

Although journals impose strict word count limits on authors when publishing abstracts, key information like randomization and blinding should be reported to the same level as the background, objectives, aims, methods, and results. If the full trial mentioned funding at the end of the trial manuscript, but not in the abstract that paper would receive a “No” against the CONSORT-A checklist. Due to the significant implications of outcomes from neuro-oncology clinical trials, declaring any funding or conflict of interest is very important to ensure transparency.

### Clinical Trial Results and the CONSORT 2010 Statement, Including CONSORT-A

The fundamental purpose of the CONSORT statement is to improve the quality of reporting and ultimately transparency of randomized trials. The CONSORT-A checklist, the abstract-specific extension of CONSORT 2010 statement was used to assess the included abstracts.

In the included trials the reporting of background, aims, and objectives, as well as what previous research supported the rationale behind the intervention was particularly high. The methodology in these trials was reported adequately and included a comprehensive description of all elements of the trial allowing transparency and easy replication of the study conditions. The trial interventions were described in detail and important variables such as dosage, route of administration, and procedures involved were present to facilitate easy replication. This theme was consistent with the included abstracts where objectives, interventions, and outcomes were well reported and with sufficient detail to facilitate replication.

In many of the included RCTs, the settings and locations of the trial were not reported. Sample size calculations are useful as it provides a metric by which readers can judge if a trial reached its planned size,^14^ was also reported poorly. None of the included trials reported on the changes to the trial outcomes. Similarly, trial status was only reported in 16 trial abstracts (44%), with authors consistently failing to mention whether the trial was ongoing, closed to recruitment, or closed to follow-up. For future trials, the addition of a sentence mentioning if any trial changes had been made and the status of the trial can remove unnecessary ambiguity and should be considered.

A key flaw in the reporting of many included trials concerned randomization. Evidence shows errors in randomization sequence generation are common and can lead to invalid conclusions if errors are not discovered.^12^ Due to this, efforts should be made to be as informative and transparent about randomization details when reporting a trial. Blinding, an important tool in protecting against potential bias was also reported poorly. This theme was also very common throughout the included abstracts, being 2 of the most poorly reported areas across both trials and abstracts. As per the CONSORT explanation and elaboration, document^10^ authors had to describe the randomization and blinding process and not merely state that randomization and blinding took place to score a “Yes” for that checklist item. While it is not our primary aim to highlight specific areas that need greater awareness across the CONSORT and CONSORT-A checklists, these 2 areas are particularly poor and prospective authors should report these details in full.

Interpretations and results of the included trials were well reported with many trials discussing how the new findings were relevant to other RCT’s. This was consistent with the included abstracts where authors consistently reported the overall results of the trial. However, although the number of participants randomized to each group were reported well across the included trials, many abstracts failed to mention this. Authors should highlight this in future abstracts to improve reporting standards.

Trial limitations were not reported in several trials. Limitations should be discussed in full, as well as any methods used to overcome said limitations. Although CONSORT-A does not encourage reporting of trial limitations, harms, and adverse effects are required to be reported. The majority of included abstracts reported important adverse effects or side effects in enough detail to receive a “Yes” on the CONSORT-A checklist. Similarly, adverse effects were well discussed in the included trials.

Trial funding was reported in most trials. Financial support, if any, should always be disclosed to allow readers to make their own judgment on whether a funding source may have influenced a result. However, in the included abstracts funding was poorly reported. When assessing the reporting quality of abstracts, funding had to be directly mentioned in the abstract to score a “Yes” against the CONSORT-A checklist.

## Limitations

This methodological review has sampled from the available neuro-oncology clinical trial literature, and is therefore not representative of all studies ever conducted. This also includes the limitation of including only articles written in the English language. We did, however, conduct a comprehensive search and included the most common pathologies studied within a trial setting. While assessment of included articles against the checklists was somewhat subjective, both data extraction and scoring were performed in duplicate by 2 review authors to minimize observer bias (J.S.S. and A.P.). Concordance was achieved at first check. The number of included protocols was relatively small, which meant that each article contributed significant weight to the mean percentage adherence score per item, so conclusive results cannot be drawn from this. However, this does highlight the infrequency of published clinical trial protocols for this health area. We used SPIRIT (2013) and CONSORT (2010) post hoc to assess all protocols, and abstracts and trials, respectively. As we included only protocols and clinical trial result articles published after 2014, both statements were technically available, although awareness at time of publication, especially when closer to the year 2014 would have been lower. We have analyzed this by dichotomizing the included clinical trial result from articles and comparing the 2 publication time ranges. This was not possible for the protocols due to limited sample size. This analysis did show improvements with time for clinical trial abstracts.

## Conclusions

The reporting quality of adult neuro-oncology clinical trial protocols and clinical trial result articles could be improved. Although more than 600 medical journals endorse CONSORT and the list of endorsers of the SPIRIT guidelines is also increasing in size, there needs to be greater awareness and possibly mandatory adherence at the time of manuscript submission, to ensure comprehensive reporting of protocols and clinical trials intended to influence practice.

## Supplementary Material

npad017_suppl_Supplementary_Data
